# Regulation of *Drosophila* hematopoietic sites by Activin-β from active sensory neurons

**DOI:** 10.1038/ncomms15990

**Published:** 2017-07-27

**Authors:** Kalpana Makhijani, Brandy Alexander, Deepti Rao, Sophia Petraki, Leire Herboso, Katelyn Kukar, Itrat Batool, Stephanie Wachner, Katrina S. Gold, Corinna Wong, Michael B. O’Connor, Katja Brückner

**Affiliations:** 1 Eli and Edythe Broad Center of Regeneration Medicine and Stem Cell Research, and Department of Cell and Tissue Biology, University of California San Francisco, San Francisco, California 94143, USA; 2 Department of Genetics, Cell Biology and Development, University of Minnesota, Minneapolis, Minnesota 55455, USA; 3 Cardiovascular Research Institute, and Helen Diller Family Comprehensive Cancer Center, University of California San Francisco, San Francisco, California 94143, USA

## Abstract

An outstanding question in animal development, tissue homeostasis and disease is how cell populations adapt to sensory inputs. During *Drosophila* larval development, hematopoietic sites are in direct contact with sensory neuron clusters of the peripheral nervous system (PNS), and blood cells (hemocytes) require the PNS for their survival and recruitment to these microenvironments, known as Hematopoietic Pockets. Here we report that Activin-β, a TGF-β family ligand, is expressed by sensory neurons of the PNS and regulates the proliferation and adhesion of hemocytes. These hemocyte responses depend on PNS activity, as shown by agonist treatment and transient silencing of sensory neurons. Activin-β has a key role in this regulation, which is apparent from reporter expression and mutant analyses. This mechanism of local sensory neurons controlling blood cell adaptation invites evolutionary parallels with vertebrate hematopoietic progenitors and the independent myeloid system of tissue macrophages, whose regulation by local microenvironments remain undefined.

## Abstract (web summary)

Hematopoietic sites in the *Drosophila* larva require the local peripheral nervous system for blood cells (hemocytes) to survive and proliferate, but how this is mediated is unclear. Here, the authors identify Activin-β from sensory neurons as signalling to hemocytes, affecting proliferation and adhesion.

In vertebrates, regulation of self-renewing blood cell populations by local organ microenvironments is poorly understood at the cellular and molecular level^1^,^2^,^3^. In a related *Drosophila melanogaster* model, blood cells (hemocytes), with similarities to vertebrate tissue macrophages and oligopotent hematopoietic progenitors, form resident clusters in segmentally repeated inductive microenvironments of the larval body wall, also known as Hematopoietic Pockets (HPs)^4^,^5^,^6^,^7^ (Fig. 1a). More than 90% of these larval hemocytes of embryonic origin are macrophage-like cells (plasmatocytes), which in the larva colonize HPs and expand by proliferation in the differentiated state^4^. Based on their functional dependence on sensory neurons of the HPs for their localization and survival, and the elevated proliferation of resident hemocytes compared to those in circulation^4^, we investigated the molecular mechanism of hemocyte induction by the sensory neurons of the peripheral nervous system (PNS).

Here we identify a molecular mechanism by which *Drosophila* hematopoiesis is controlled by the PNS. Activin-β (Actβ, Act), a Transforming Growth Factor-β (TGF-β) family ligand, is specifically produced by multidendritic neurons and chordotonal organs of the PNS, and acts to regulate the proliferation and long-term adhesion of hemocytes. PNS activation by agonist treatment drives expansion of the blood cell pool, while specific silencing of sensory neurons affects resident hemocyte number and localization. Actβ plays a key role in this regulation, as evidenced by the induction of *Actβ* expression in response to PNS activity, and a blunted response in *Actβ* mutants. These findings shed new light on the mechanisms by which local microenvironments regulate blood cell adaptation and may integrate sensory inputs.

## Results

### Sensory neurons form an interface with the blood cell system

**Figure 1:**
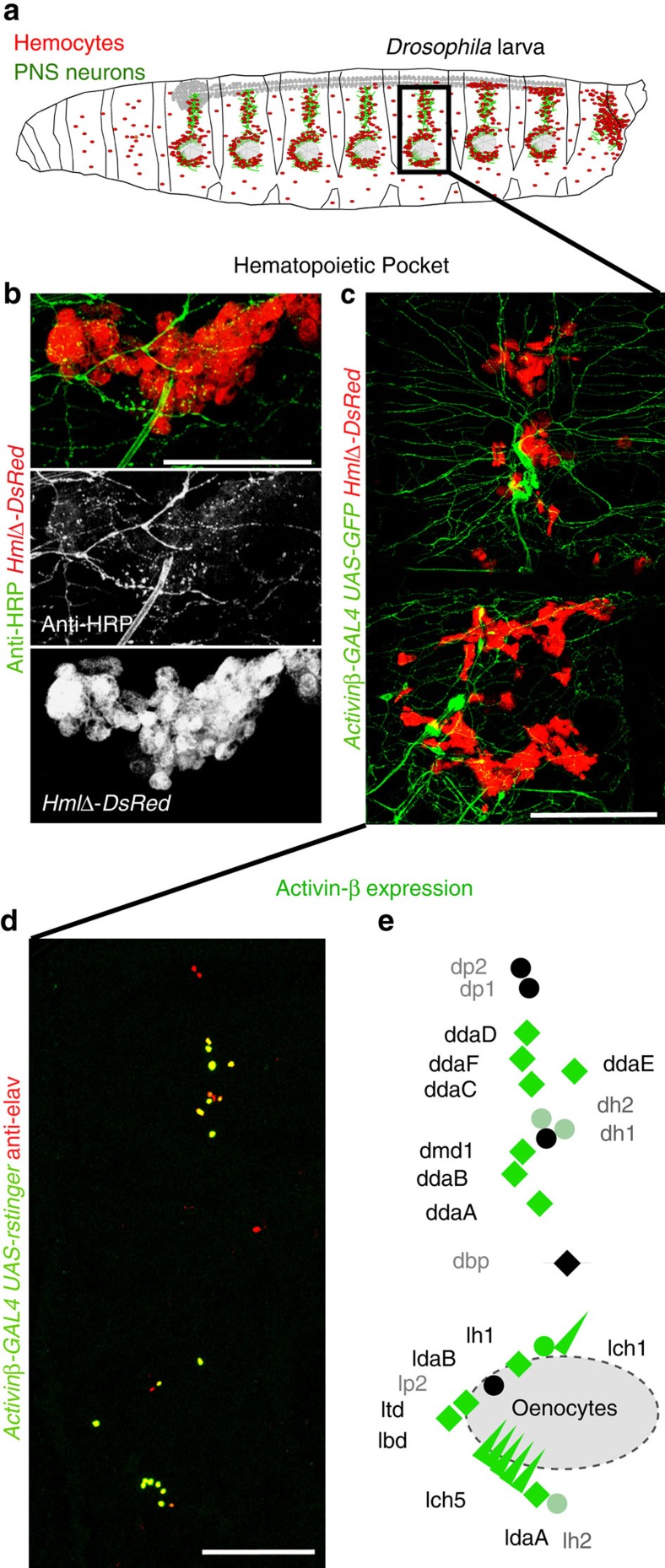
PNS sensory neurons produce Activin-β and are surrounded by hemocytes. (**a**) Model of a *Drosophila* larva; hemocytes in red, neurons in green. Boxed area marks a HP. (**b**) Close-up of a lower part of a HP illustrating intricate neuronal extensions in areas of hemocytes; neurons (Anti-HRP, green), hemocytes (*HmlΔ-DsRed*, red), lateral patch area. Middle and lower panel show single channels. (**c**) Actβ expressing larval PNS neurons marked by reporter *Actβ-GAL4; UAS-mCD8GFP* (green), Actβ positive neurons colocalize with hemocytes marked by *HmlΔ-DsRed* (red). Fillet prep containing all tissue layers. (**d**) Actβ expression pattern in larval PNS neurons, *Actβ-GAL4/+; UAS-r-stinger/+* (green), pan-neuronal anti-elav (red). Fillet prep containing all tissue layers. (**e**) Model of Actβ expression (green) in most multidendritic neurons (diamonds), chordotonal organs (triangles), and some external sensory neurons (circles). Scale bars, **b**, 50 μm; **c**,**d**, 100 μm.

First we examined the local anatomy of PNS neurons and resident (sessile) hemocytes. High magnification imaging revealed that PNS neurons form intricate extensions to areas of resident hemocytes (Fig. 1b), suggesting an interface that allows direct neuron-to-hemocyte communication. Using a split GFP approach, GFP Reconstitution Across Synaptic Partners (GRASP)^8^,^9^ (Supplementary Fig. 1a), we confirmed that hemocytes are anatomically extremely close, and potentially form direct contacts with PNS neurons and glia (Supplementary Fig. 1b–e). To identify specific molecular signals that mediate this communication, we screened components of several key signalling pathways utilizing *in vivo* RNA interference (RNAi)^10^, searching for defects in hemocyte number and/or localization. Based on this, we focused on the role of the TGF-β family-related dSmad2 (Smox) pathway^11^ in hemocyte regulation. To identify the responsible ligand, we examined expression of the putative pathway ligands *Activin-β (Actβ), Dawdle (daw)* and *myoglianin (myo)* using ligand GAL4 reporters (O’Connor lab and^12^,^13^). *Actβ* was highly expressed by specific subsets of sensory neurons in the HPs, in particular the multidendritic (md) neurons and chordotonal organs^14^ (Fig. 1d,e). None of the other ligands showed obvious expression in the sensory neurons. Colabelling confirmed localization of *Actβ*-producing neurons with resident hemocytes in the HPs (Fig. 1c). In contrast, *Actβ* was not detectably expressed in other components of the HPs, such as epidermis, muscle and oenocytes, (Figs 1c,d, 6c,d,f,g). *Actβ* was also highly expressed in motor neurons and other neurons of the central nervous system (CNS) as described previously, similar to related ligands of the TGF-β family^11^,^15^,^16^,^17^,^18^,^19^,^20^. However, the vast majority of hemocytes in the larva, present as resident hemocytes in the HPs, were physically separated from the CNS, motor neurons and axon terminals of motor neurons (Supplementary Fig. 2a,b, and see anatomical description of the HPs in ref. 4).

### Actβ signalling promotes blood cell proliferation and adhesion

**Figure 2:**
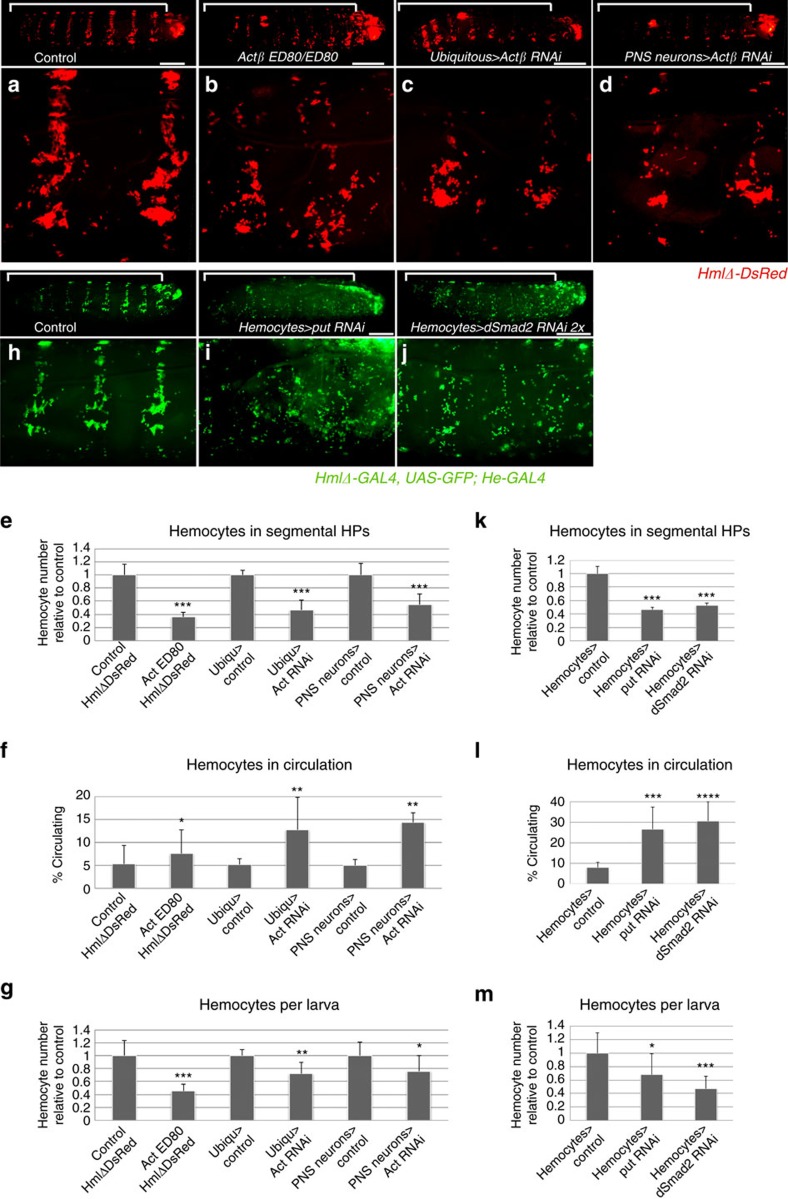
Loss of Activin-β/dSmad2 signalling results in hemocyte defects. (**a**–**d**) Loss of Actβ in neurons affects hemocytes, lateral view of larvae (top panels) with close-up (lower panels). (**a**) Control *HmlΔ-DsRed* /+ (**b**) *Actβ* mutant, *HmlΔ-DsRed/+; Actβ*^*ED80/ED80*^. (**c**) *Actβ* RNAi silencing with ubiquitous driver *Actin5C-GAL4*. (**d**) *Actβ* RNAi (*UAS-Act RNAi* Vienna GD) silencing in PNS neurons, md neuron specific driver *21-7-GAL4*. (**e**) Hemocyte counts in segmental HPs (bracketed areas in **a**–**d** top panels), *n*=4–7 per genotype. (**f**) Fraction of circulating hemocytes, *n*=4 to 8 per genotype. (**g**) Total hemocyte counts per larva, *n*=4 to 8 per genotype. In **e**–**g** experiment numbers relative to matching control cohorts, genotypes *HmlΔ-DsRed/+; Actβ*^*ED80/ED80*^ (matching control: *HmlΔ-DsRed/+); Actin5C-GAL4, HmlΔ-DsRed/+; UAS-Actβ* RNAi/+ (matching control: *Actin5C-GAL4, HmlΔ-DsRed/+)*; *21-7-GAL4, UAS-mCD8GFP, HmlΔ-DsRed/+; UAS-Actβ* RNAi*/+* (matching control: *21-7-GAL4, UAS-mCD8GFP, HmlΔ-DsRed/+).* Hemocyte numbers were assessed in larvae 64–75 h AEL (2.2–2.6 mm) (**h**–**j**) Hemocyte-specific RNAi silencing of Actβ pathway components, driver *HmlΔ-GAL4, UAS-GFP; He-GAL4*. Top panels whole larva lateral view, lower panels closeups. (**h**) Control; (**i**) *UAS-put* RNAi; (**j**) *UAS-dSmad2* RNAi. (**k**) Hemocyte counts in segmental HPs (bracketed areas **h**-**j** in top panels), *n*=4 to 7 per genotype. (**l**) Fraction of circulating hemocytes, *n*=7 to 10 per genotype. (**m**) Total hemocyte counts per larva, *n*=7 to 11 per genotype; larvae 53–60 h AEL (1.8–2.0 mm). In (**k**–**m**) experiment numbers relative to matching control cohorts are shown; genotypes *HmlΔ-GAL4, UAS-GFP/+; He-GAL4/+* (control); *HmlΔ-GAL4, UAS-GFP/+; He-GAL4/UAS-put RNAi* or *HmlΔ-GAL4, UAS-GFP/UAS-dSmad2 RNAi; He-GAL4/+* (experiment). Scale bars:, **a**–**d** and **i**,**j** 0.5 mm. Error bars represent s.d., and two-tailed *t*-test values correspond to NS (not significant) *P*>0.05; *P≤0.05; **P≤0.01; ***P≤0.001; ****P≤0.0001.

Next we investigated the effects of Actβ/dSmad2 pathway loss- and gain of function (lof, gof). Studying the viable null mutant *Actβ*^*ED80*^ (ref. 21), or silencing *Actβ* by *in vivo* RNAi, driving transgene expression ubiquitously or in sensory md neurons, we found diminished hemocyte numbers in the segmental HPs (Fig. 2a–e). Consistent with this, we observed an increase in the fraction of circulating hemocytes (Fig. 2f), and overall reduced total hemocyte numbers per larva (Fig. 2g), using a method of quantitative hemocyte retrieval from single *Drosophila* larvae^22^. These defects resembled hemocyte phenotypes seen on PNS neuron ablation^4^ and suggested defects in hemocyte adhesion, proliferation and/or survival. Silencing of *Actβ* in motor neurons using the driver *OK6-GAL4* (Supplementary Fig. 2d,e,h,i) did not affect the localization of hemocytes in the HPs (Supplementary Fig. 2f,g,j,k), and resulted only in minor reductions of total hemocytes during various time points of larval development, which were non-significant according to Student’s *t*-test (Supplementary Fig. 2l). *Actβ*^*ED80*^ mutants showed partial penetrance (62% mutant phenotype) and were analysed side-by-side with controls as 2nd instar larvae, to avoid compensatory mechanisms that became evident in 3rd instar larvae. In all backgrounds of *Actβ* lof, PNS neuron clusters were present and appeared normal by cell number and dendritic morphology. This suggested a role for Actβ signalling in hemocytes, rather than indirect effects due to roles of Actβ in the nervous system^11^,^15^,^18^,^19^.

To substantiate the role of Actβ/dSmad2 signalling in hemocytes, we systematically determined the effects of hemocyte-autonomous RNAi silencing of the Activin type II receptor *punt (put)*^23^ and the signal transducer *dSmad2* (ref. 11). Similar to the loss of Actβ in the microenvironment, knockdown of Actβ pathway components in hemocytes resulted in diminished numbers of resident hemocytes in the segmental HPs (Fig. 2h–k). Silencing of the Activin type I receptor *baboon (babo)*^11^ showed similar albeit milder effects on hemocyte localization. Silencing of the pathway in hemocytes by knockdown of *put* or *dSmad2* further resulted in increased fractions of circulating hemocytes (Fig. 2l) and reduced total hemocyte numbers per larva (Fig. 2m). With prolonged RNAi silencing of *put* and *dSmad2*, the reduction in total hemocytes eventually reversed in older larvae (Supplementary Fig. 3), again implying putative compensatory mechanisms that take effect in the prolonged absence of dSmad2 signalling. Silencing of *put* and *dSmad2* under the control of *HmlΔ-GAL4* had no effect on lymph gland hemocytes in larvae of the developmental window studied (Supplementary Fig. 4a–i), and no concomitant increase in the fraction of crystal cells, neither of the lymph gland nor the embryonic lineage of hemocytes, was observed (for lymph gland see Supplementary Fig. 4c,f,i). This was examined because in the larva, plasmatocytes are known to give rise to small numbers of crystal cells^24^,^25^. Taken together, our findings suggest that sensory neuron-produced Actβ signals through the dSmad2 pathway in hemocytes, which supports hemocyte numbers and promotes hemocyte localization to the HPs.

**Figure 3:**
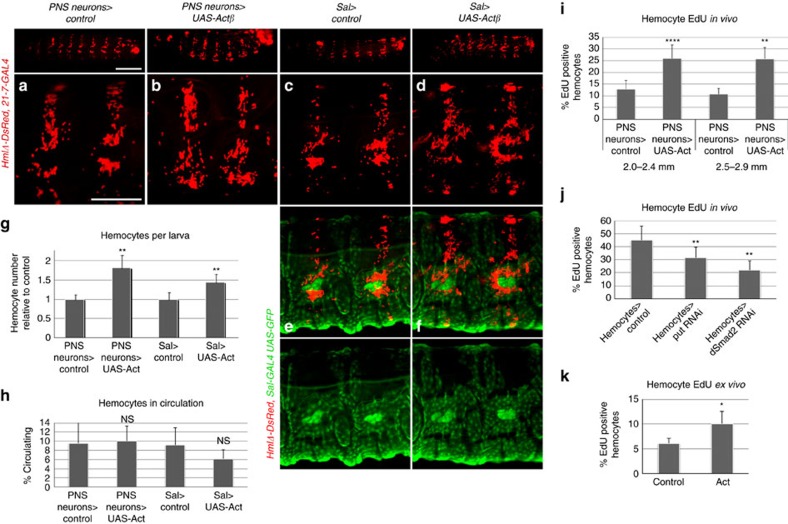
Activin-β promotes hemocyte proliferation. (**a**,**b**) Control (*HmlΔ-DsRed* (red), and overexpression of Actβ in PNS neurons by *21-7-GAL4* with hemocytes *HmlΔ-DsRed* (red). Whole larvae lateral view (top panel), and closeup (lower panel). (**c**,**d**) Control and ectopic expression of Actβ in ventral epidermis and oenocytes using the driver *Sal-GAL4* (*GMR85E05-GAL4)* marked by *UAS-GFP* (green), and hemocytes *HmlΔ-DsRed* (red). Whole larvae lateral view (top panel), and closeup (lower panel). (**e**,**f**) Same as in (**c**,**d**) but combined (red, green) and single channels (green) of close-up images shown. (**g**) Total hemocyte counts per larva, genotypes are *21-7-GAL4, UAS-mCD8GFP, HmlΔ-DsRed/+; UAS-Actβ/+ (*control is *21-7-GAL4, UAS-mCD8GFP, HmlΔ-DsRed/+), n*=4, seen with two different transgenes (*n*=8 total); and *HmlΔ-DsRed/+; Sal-GAL4, UAS-GFP/UAS-Actβ,* (control is *HmlΔ-DsRed/+; Sal-GAL4, UAS-GFP/+*), *n*=5 to 6. (**h**) Hemocytes in circulation, genotypes and n as in **g**. (**i**) *In vivo* EdU incorporation, genotypes *21-7-GAL4, UAS-mCD8GFP, HmlΔ-DsRed/+* (control) and *21-7-GAL4, UAS-mCD8GFP, HmlΔ-DsRed/+*; *UAS-Actβ (III)/+*; *n*=36 for larvae 60–70 h AEL (2.0–2.4 mm), and *n*=8 for larvae 71–83 h AEL (2.5–2.9 mm). (**j**) *In vivo* EdU incorporation, *HmlΔ-GAL4, UAS-GFP; He-GAL4* x *yw* (control); x *UAS-put* RNAi and *UAS-dSmad2* RNAi (experiment larvae); *n*=6; larvae 46–52 h AEL (1.5–1.7 mm). (**k**) EdU incorporation of released larval hemocytes stimulated with Actβ *ex vivo*; genotype *HmlΔ-GAL4, UAS-GFP; He-GAL4*; average over 4 independent experiments (Actβ +/− conditioned media from 2 cell lines in 2 replicates each). Scale bars, **a** (upper panel), 0.5 mm; **a** (lower panel), 0.2 mm. Error bars represent s.d., and two-tailed *t*-test values correspond to NS (not significant) *P*>0.05; *P≤0.05; **P≤0.01; ***P≤0.001; ****P≤0.0001.

To determine whether Actβ/dSmad2 signalling has trophic and/or proliferative roles in hemocytes, we focused on the effects of *Actβ* overexpression and *babo* gain of function. Moderate *Actβ* overexpression in PNS neurons, or ectopic sites in oenocytes and epidermis using the driver *Spalt (Sal)-GAL4*, resulted in increased total hemocyte numbers per larva (Fig. 3a–g), while silencing of *Actβ* in these locations had no significant effect according to Student’s *t*-test (Supplementary Fig. 5a–c). The increase in total hemocyte numbers on *Actβ* overexpression was accompanied by increased *in vivo* EdU incorporation, suggesting enhanced hemocyte proliferation (Fig. 3i). Consistently, silencing of *put* and *dSmad2* in hemocytes resulted in reduced EdU incorporation of hemocytes *in vivo* (Fig. 3j). To substantiate a direct role of Actβ in hemocyte proliferation, we also examined larval hemocytes *ex vivo* under conditions of Actβ stimulation. Indeed, we found that Actβ promoted EdU incorporation, indicative of increased hemocyte proliferation (Fig. 3k). In contrast, high overactivation of the pathway by hemocyte specific expression of the constitutively activated receptor *babo-CA* resulted in reduced hemocyte numbers per larva (Supplementary Fig. 6a–c) and drove hemocytes into apopotosis (Supplementary Fig. 6d). Neither *Actβ* overexpression nor dSmad2 pathway silencing in hemocytes increased the rate of hemocyte apoptosis (Supplementary Fig. 6c,d). Based on this, we conclude that the level of Actβ/dSmad2 signalling may determine the nature of the hemocyte response. At moderate activation levels, Actβ/dSmad2 signalling is a positive regulator of hemocyte number that promotes proliferation, while high overactivation of the pathway drives cells into apoptosis. Consequently, we anticipate that the amplitude of *Actβ* expression is likely to be tightly regulated.

**Figure 4:**
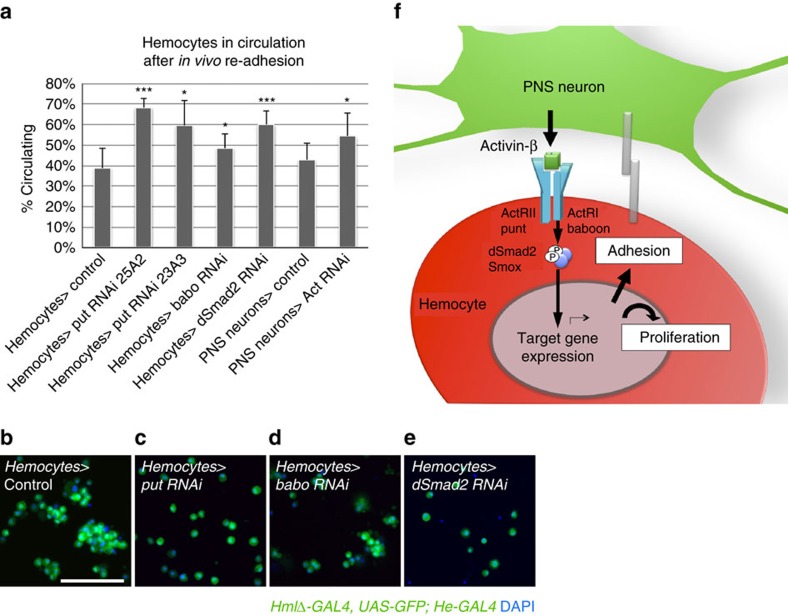
Activin-β promotes hemocyte adhesion. (**a**) *In vivo* re-adhesion of hemocytes after mechanical disturbance, bars illustrate percent of circulating hemocytes; genotypes are *HmlΔ-GAL4, UAS-GFP; He-GAL4* × UAS-RNAi transgenes and control; and *21-7-GAL4, UAS-mCD8GFP, HmlΔ-DsRed* × *UAS-Actβ RNAi* and control; *n*=7 to 8. (**b**–**e**) *Ex vivo* released hemocytes show different levels of aggregation; *HmlΔ-GAL4, UAS-GFP; He-GAL4* × (**b**) Control; (**c**) *UAS-put* RNAi; (**d**) *UAS-babo* RNAi; (**e**) *UAS-dSmad2* RNAi. (**f**) Model of Actβ production by sensory neurons and induction of hemocyte responses in proliferation and adhesion. Scale bars, **b**, 50 μm. Error bars represent s.d., and two-tailed *t*-test values correspond to NS (not significant) *P*>0.05; *P≤0.05; **P≤0.01; ***P≤0.001; ****P≤0.0001.

Next we examined the role of Actβ/dSmad2 signalling in hemocyte localization. Ectopic expression of *Actβ* in areas typically devoid of hemocytes, such as the *Sal* expressing ventral areas of the epidermis and oenocytes (Fig. 3e,f) or imaginal discs, did not result in a uniform adhesion or attraction of hemocytes, i.e., in the alternating gap areas of the epidermis where no sensory neuron clusters are located (Fig. 3e,f). Further, uniform overactivation of the pathway by hemocyte expression of *babo-CA* showed a largely unaffected overall pattern of resident hemocytes, despite the above-mentioned apoptosis of hemocytes (Supplementary Fig. 6a,b). This argued against a function of Actβ in hemocyte chemoattraction by gradient formation and led us instead to focus on a potential role of Actβ in the induction of hemocyte adhesion. Ectopic expression of *Actβ* produced an overall trend of decreasing the fraction of circulating hemocytes, although this effect remained statistically insignificant by *t*-test (Fig. 3h), and accumulation of hemocytes in ectopic areas seemed minimal (Fig. 3c–f). Seeking a more sensitive assay to quantify hemocyte adhesion, we took advantage of the fact that resident hemocytes of the HPs can be mobilized by mechanical disturbance and spontaneously re-adhere to the body wall within 30–45 min (ref. 4). Using an established protocol for this assay^22^, we examined the adhesive properties of hemocytes under various Actβ/dSmad2 pathway conditions. Indeed, dSmad2 pathway knockdowns in hemocytes, or *Actβ* silencing in PNS neurons, diminished hemocyte re-adhesion as evidenced by increased fractions of circulating hemocytes (Fig. 4a). However, dSmad2 pathway activation did not promote increased re-adhesion, suggesting an additionally needed, rate-limiting step in hemocyte adhesion. Moreover, we found that hemocyte-autonomous knockdowns of dSmad2 pathway components, but not *Actβ* silencing in neurons, produced a lack in hemocyte cluster formation and self-adhesion (Fig. 4b–e). Taken together, we conclude that the Actβ/dSmad2 pathway directly or indirectly promotes hemocyte adhesion to the microenvironment (Fig. 4f, Model), a process that we predict requires in addition another, rate-limiting step. dSmad2 signalling may have an additional autonomous roles in hemocyte clustering/self-adhesion, which is revealed only when the pathway is blocked in hemocytes, thereby excluding alternative signalling by other Act family ligands as might be the case in *Actβ* lof backgrounds.

### *Actβ* links sensory neuron activity with blood cell responses

**Figure 5:**
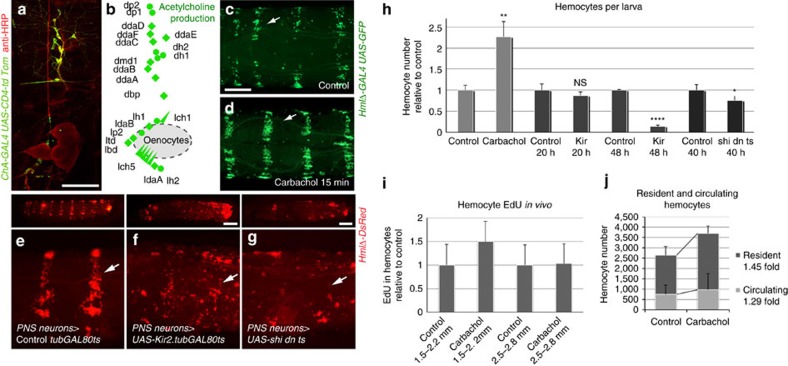
PNS neuron activation promotes hemocyte recruitment and proliferation. (**a**) Labelling of cholinergic PNS neurons using the diagnostic driver *Cha-GAL4* x *UAS-CD4-td Tom* and anti-HRP 488; false colouring of ChA positive neurons in green, HRP in red. (**b**) Model, Cha-GAL4 positive signal in all PNS neurons. (**c**,**d**) Stimulation of PNS neurons. Live imaging of larvae marked with *HmlΔ-GAL4, UAS-EGFP*, dorsal view. (**c**) Control; (**d**) carbachol, short-term exposure (10 mgml^−1^, 15 min). (**e**–**g**) Live imaging of hemocytes (*HmlΔ-DsRed*, red) after transient disturbance of electrochemical signalling in PNS neurons, hemocytes in red; arrows point to HPs. Larvae lateral view (top panels), closeups (lower panels). (**e**) Control, *21-7 GAL4, UAS-mCD8GFP, HmlΔ-DsRed/+*; (**f**) *Kir2.1* expression to hyperpolarize neurons, *21-7 GAL4, UAS-mCD8GFP, HmlΔ-DsRed/ tubGAL80^ts^; UAS-Kir2.1/+*; (**g**) Expression of *UAS-shi dn*^*ts*^ in neurons, *21-7 GAL4, UAS-mCD8GFP, HmlΔ-DsRed/+; UAS-shi dn*^*ts*^*/+*. (**h**) Larvae −/+ carbachol exposed from hatching onward, and larvae −/+ genetic neuronal silencing as in (**f**) and (**g**), heats shocks to induce transgenes were applied as indicated; total hemocyte counts from single larvae (2nd instars, 66–78 h AEL corresponding to 2.3–2.7 mm). For each experimental cohort, total hemocyte numbers of experiment sets relative to side-by-side control sets of larvae are shown; *n*=3 to 5 and comparable effect in independent repeats. (**i**) *In vivo* EdU incorporation of hemocytes from larvae exposed to −/+ carbachol (0.7 mg ml^−1^) from 1st instar (∼22 h AEL) onward. Genotype is *HmlΔ-GAL4, UAS-GFP; He-GAL4;* larval sizes as indicated, corresponding to 2nd instar 47–65 h AEL (1.5–2.2 mm) and 72–80 h AEL (2.5–2.8 mm). EdU incorporation of experiment sets relative to side-by-side control sets of larvae are shown; average of three independent experiments, total *n*=6 to 9 per condition. (**j**) Fraction of resident and circulating hemocytes of −/+ carbachol treated larvae 74–85 h AEL (2.6–3.0 mm), on carbachol (0.7 mg ml^−1^) since first instar (∼22 h AEL); genotype *HmlΔ-GAL4, UAS-GFP; He-GAL4*. Carbachol treated animals show a larger increase in the resident than in the circulating population (1.45 fold versus 1.29 fold); *n*=13 to 14 per condition. Scale bars, **a**, 100 μm; **c**, 0.2 mm; and **f**,**g**, 0.5 mm. Error bars represent s.d., and two-tailed *t*-tests value correspond to NS (not significant) *P*>0.05; *P≤0.05; **P≤0.01; ***P≤0.001; ****P≤0.0001.

Considering the anatomical contacts of PNS neurons with hemocytes, and the role of Actβ as neuron-emanating signal that supports hemocyte proliferation and adhesion, we sought to determine whether PNS neuron activity may regulate blood cell behaviours. Using a diagnostic driver for acetylcholine production, *Cha-GAL4*, we confirmed previous reports that PNS neurons are cholinergic (Fig. 5a,b)^26^. This allowed us to use the pan Acetylcholine Receptor (AChR) agonist carbachol (carbamoylcholine) for the stimulation of larval sensory neurons. Interestingly, we found that cuticle exposure of intact larvae to carbachol promoted short-term recruitment of hemocytes to the HPs within 15 min, resulting in an enhanced resident hemocyte pattern (Fig. 5c,d). Since carbachol may not target neurons of the PNS only, we confirmed our findings by genetic manipulation of the PNS. Using the PNS neuron-specific driver *21-7-GAL4* that is nearly devoid of CNS expression, we transiently silenced sensory neurons using two methods; (1) hyperpolarization through transient expression of the inward rectifying K^+^ channel *Kir2.1* (ref. 27), or (2) transient interference with neuronal endocytosis and synaptic vesicle recycling through expression of the temperature-sensitive dominant-negative dynamin *UAS-shi dn*^*ts*^ (ref. 28). Indeed, both sensory neuron manipulations strongly and reversibly disturbed the pattern of larval resident hemocytes within a short-term 2 h time frame, indicative of compromised hemocyte adhesion to the HPs (Fig. 5e–g). As a control, we expressed the same transgenes in glia using *repo-GAL4* (ref. 29), yet no hemocyte phenotypes were observed (Supplementary Fig. 7a–d), demonstrating specificity of the observed hemocyte responses to neuronal silencing.

In addition to these short-term (15 min–2 h) effects on hemocyte localization to the HPs, we examined the long-term effects of stimulation by carbachol, or neuronal silencing by *Kir2.1* or *UAS-shi dn*^*ts*^, on hemocytes. Interestingly, we found that carbachol exposure over several days of larval development significantly increased total hemocyte numbers per larva compared to controls according to Student’s *t*-test (Fig. 5h). Consistently, silencing of PNS neurons through various regimens of heat shock induction of *Kir2.1* or the dominant-negative dynamin *shi dn*^*ts*^ over 20–48 h had opposite effects, resulting in larvae with reduced total hemocyte numbers (Fig. 5h). To examine whether the carbachol-induced increase in hemocyte number was based on proliferation, we quantified *in vivo* EdU incorporation of hemocytes. Indeed, carbachol-exposed larvae showed about 1.5-fold increased EdU incorporation over controls, an effect which interestingly was mainly seen in younger 2nd instar larvae (Fig. 5i). Further, carbachol-exposed larvae showed an overall larger increase in the resident hemocyte population of the hematopoietic sites compared to the circulating fraction (Fig. 5j), consistent with our model proposing enhanced proliferation of resident hemocytes in the HPs. Taken together, our data suggest that PNS neuronal activity supports hemocyte localization and expansion by proliferation, and predict a molecular signal from PNS neurons that is inducible by sensory activity.

**Figure 6:**
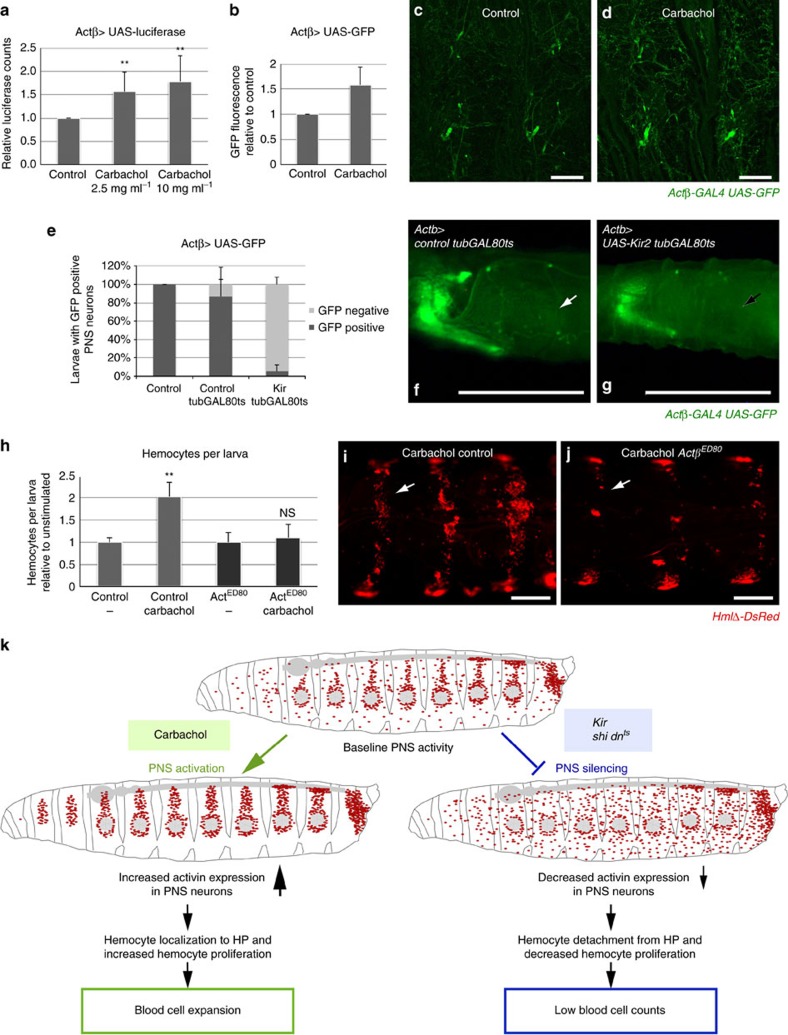
Neuronal activity promotes Actβ expression which drives hemocyte expansion. (**a**–**d**) Carbachol induces dose-dependent transcriptional induction of an Actβ reporter. (**a**) Quantification of luciferase expression in larval fillets of *Actβ-GAL4, UAS-CD4-tdGFP/UAS-luciferase* animals, stimulated by increasing doses of carbachol as intact larvae (2.5 mgml^−1^; 10 mgml^−1^). Pools of 2-3 larval fillets per sample in biological replicates; carbachol-stimulated conditions were normalized to unstimulated condition (total *n*=4–6 per condition); statistics show average of four such normalized independent experiments. (**b**) Carbachol-induced increase of GFP expression in fillets of *Actβ-GAL4, UAS-CD4-tdGFP* larvae, signal quantification over three independent sets of images, normalized to unstimulated condition and averaged (*n*=3). (**c**,**d**) Sample set of images as in **b**. (**c**) Control, (**d**) 4 h stimulation with carbachol as intact larva, 2.5 mg ml^−1^. (**e**–**g**) Transient PNS silencing with *UAS-Kir2.1* reduces GFP expression; induction for 22 h at 27 °C. (**e**) Fraction of larvae showing visible GFP expression in PNS neurons, genotype *tubGAL80^ts^/+; Actβ-GAL4, UAS-CD4-tdGFP*/*+* (control) and *tubGAL80^ts^/+; Actβ-GAL4, UAS-CD4-tdGFP*/*UAS-Kir 2.1* (experiment); *n*=20 to 22, average of 2 independent biological replicates. (**f**) Sample images of control and (**g**) Kir2.1 expressing larva. Note that Kir2.1 coexpression reduces GFP expression in PNS and CNS; CNS on left side (anterior). (**h**) Comparison of hemocyte expansion in *Actβ*^*ED80*^ mutant larvae and controls, genotypes *HmlΔ-DsRed/+; Actβ*^*ED80/ED80*^ and control *HmlΔ-DsRed/+.* Total hemocyte counts per larva of carbachol-treated cohorts (0.7 mg ml^−1^) and control food cohorts; *n*=6–8 per genotype and condition. (**i**,**j**) Comparison of control and *Actβ*^*ED80*^ mutant in the recruitment of hemocytes to HPs after short-term carbachol exposure (10 mg ml^−1^, 15 min), genotypes as in **h**, dorsal view. (**k**) Model. Actβ in sensory neuron-induced blood cell adaptation. PNS neuronal activity triggered by carbachol elevates Actβ expression in neurons, resulting in enhanced hemocyte proliferation and localization to HP. Neuronal silencing has opposite effects. Scale bars, **c**,**d**, 100 μm; **f**,**g**, 0.5 mm; **i**,**j**, 0.2 mm. Error bars represent s.d., and two-tailed *t*-test values correspond to NS (not significant) *P*>0.05; *P≤0.05; **P≤0.01; ***P≤0.001; ****P≤0.0001.

To determine whether Actβ might play a role as this inducible signal, we quantified *Actβ* expression in sensory neurons of varying states of activity. Indeed, exposure of intact *Drosophila* larvae to carbachol induced a substantial and dose-dependent increase in *Actβ* expression within 2–3 h, as quantified by the expression of a *UAS-luciferase* transgene driven by the *Actβ-GAL4* reporter (Fig. 6a). For this experiment, we focused on reporter expression in the PNS by carefully removing the CNS before luciferase quantification. To examine whether the increase in *Actβ-GAL4* expression was specific to sensory neurons, we co-expressed a *UAS-GFP* transgene. GFP imaging of freshly dissected larvae confirmed increased expression in PNS neurons, and did not show signs of ectopic expression in unrelated tissues (Fig. 6b–d). In a converse experiment, we examined whether inducible expression of *Kir2.1* affects expression of *UAS-GFP* by the reporter *Actβ-GAL4* in PNS neurons. Inducing expression (through *tub-GAL80^ts^)* in limited time windows of 22 h to visualize GFP expression yet avoiding lethality by high expression of *Kir2.1*, we observed induction of GFP in *Actβ* expressing PNS neurons of controls, while this GFP signal was largely absent in parallel cohorts silenced by coexpressed *Kir2.1* (Fig. 6e–g). In addition to its effects on the PNS, *Kir2.1* coexpression also reduced *Actβ-GAL4* driven GFP expression in the CNS (Fig. 6f,g).

Finally, we asked whether Actβ is required for the induction of hemocyte responses on stimulation with carbachol. Comparing *Actβ*^*ED80*^ null mutant larvae with Actβ-competent controls, we found that loss of *Actβ* attenuated the effect of carbachol-induced blood cell expansion (Fig. 6h). This suggested that Actβ plays a major role in the cholinergic regulation of hemocytes, consistent with a model of neuronal activity-induced Actβ expression (Fig. 6k). However, *Actβ* mutants showed mild albeit by *t*-test insignificant carbachol-induced blood cell expansion (Fig. 6h) and partial short-term hemocyte recruitment to the HPs on 15 min of carbachol exposure (Fig. 6i,j), suggesting additional inducible signal/s that may contribute to these effects. Taken together, our findings support a model in which PNS neuronal activity promotes Actβ expression, which in turn drives hemocyte expansion and long-term localization to the HPs (Fig. 6k).

## Discussion

This research identified *Actβ* as one of the elusive genes that govern hemocyte proliferation in the hematopoietic sites (HPs) of the *Drosophila* larva, as was predicted by previous functional studies^4^. Our work further links *Actβ* RNA expression to the level of PNS neuronal activity. This model implies that increased expression of *Actβ* would give rise to higher levels of active Actβ protein, although the formal demonstration awaits development of a suitable tool for the detection of Actβ protein. In the future, it will be interesting to study specific sensory stimuli that trigger hemocyte responses. Sensory neurons of the PNS have a prime function in detecting innocuous and noxious sensory stimuli such as mechanical strain, temperature, chemicals and light^30^,^31^, many of which signal potentially harmful conditions that may cause tissue damage. Thus, linking the detection of challenging conditions with the adaptive expansion of the blood cell pool may be an efficient system to elevate the levels of macrophages, to remove and repair damaged tissues, enhancing the overall fitness of the animal. Because *Drosophila* larval hemocytes persist into the adult stage^6^,^7^, the mechanism of sensory neuron-induced blood cell responses may allow adaptation of the animal beyond the larval stage.

In *Drosophila* self-renewing hemocytes, Actβ/dSmad2 signalling has diverse effects on proliferation, apoptosis and adhesion. Our *ex vivo* data indicate that hemocyte proliferation is likely a direct effect, which is consistent with similar roles of babo/dSmad2 in other tissues such as *Drosophila* imaginal discs and brain^11^,^21^, and TGf-β family dependent proliferation in vertebrate systems^32^,^33^. Echoing our findings of *babo-CA* driven hemocyte apoptosis, TGF-β family mediated direct or indirect effects on apoptosis have been described in invertebrate and vertebrate systems^33^,^34^. Overall, TGF-β family signalling is known for its multifaceted biological roles, depending on the cellular contexts and levels of ligand stimulation, which often translate into qualitatively distinct transcriptional and other cellular responses, that are mediated by both Smad and non-Smad signalling mechanisms^32^,^33^. While *Drosophila* Actβ and possibly related TGF-β family ligands are known to signal through the induction of *ecdysone receptor (EcR)* in some but not all *Drosophila* tissues^15^,^35^, we found no indication for a link with EcR expression in hemocytes, suggesting other signalling mechanisms in the regulation of larval blood cell responses. In the studied *Drosophila* system, it further remains to be seen whether Actβ/dSmad2 signalling has direct or indirect effects on hemocyte adhesion, and which other rate-limiting step/s may contribute to this process. Since hemocyte-autonomous loss of dSmad2 signalling causes a more severe phenotype than *Actβ* lof, we speculate that other Act family ligands such as *daw* and *myo*, which are expressed in various tissues including surface glia, muscle, fat body, gut, and imaginal discs^11^,^17^,^20^,^36^,^37^ may partially substitute for *Actβ* in its absence. Overall, Actβ is likely to be only one player in a more complex regulatory network. Future research will identify other inducible signals from neurons that regulate neuron-blood cell communications. This is predicted from *Actβ* mutants that only partially block carbachol-induced blood cell responses. *Actβ/dSmad2* lof and pathway silencing in hemocytes also reveal an underlying ability of the cells to compensate for the lack of this signalling pathway and the associated impairment in proliferation. Time course experiments with various RNAi lines suggest that the amplitude and temporal occurrence of the compensatory response may be proportional to the severity of the block in dSmad2 signalling. Future investigation will address whether the related BMP/Mad pathway might play a part in this, as silencing of *Mad* in hemocytes appeared to dampen elevated hemocyte numbers seen in *dSmad2* null mutants. Similar observations of *dSmad2* lof causing Mad overactivation have been reported in the *Drosophila* wing disc and neuromuscular junction previously^20^,^38^.

Larval development may comprise distinct sensitive phases for the regulation of hemocyte responses. This is supported by carbachol promoting hemocyte proliferation preferentially in the early-mid 2nd instar larva, that is, at a stage when hemocytes are still tightly localized to the HPs^4^,^22^. Likewise, the effects of *Actβ* lof and pathway silencing in hemocytes are more pronounced in younger larvae, suggesting a possible stronger dependence on the pathway, in addition to the emergence of compensatory mechanisms under lof conditions over time (above). Moreover, it will be interesting to investigate whether Actβ signalling may not only vary temporally, but also by the ability of cell types to produce active Actβ ligand, thereby influencing signalling outcomes, consistent with the cell type specific processing known for Activins and other ligands of the TGF-β family in both invertebrates and vertebrates^16^,^39^,^40^.

*Drosophila* Actβ has previously been studied for its role in the formation and function of neuromuscular junctions in the *Drosophila* larva, where Actβ expressing motor neurons project axons from the CNS, reaching from the center of the larva to the muscle layers of the body wall^16^,^17^,^20^ However, resident hemocytes are shielded from these areas through the muscle layers of the body wall, which also form the base of the HPs, thereby creating an anatomical space between the muscle layers and epidermis^4^ where resident hemocytes and Actβ expressing sensory neurons colocalize (i.e., the Hematopoietic Pocket). The model that sensory neurons signal to adjacent hemocytes in the HPs is further supported by the fact that *Actβ* silencing in motor neurons did not affect resident hemocyte localization and had, by *t*-test, no significant effect on hemocyte numbers. However, we cannot completely rule out involvement of alternative or additional scenarios, for example, that experimental manipulations of PNS activity, which also feed back to the CNS, would in turn trigger a signal to motor neurons that may respond by secreting Actβ and/or another factor/s, thereby influencing hemocytes and/or the PNS itself. Likewise, although we confirmed the direct effect of Actβ on hemocytes *ex vivo*, and found no signs of altered sensory neuron morphology under *Actβ* lof/silencing, we cannot rule out that in the larva, Actβ may contribute to molecular changes in the PNS that in turn might contribute to the observed hemocyte effects.

Sensory neurons of the HPs project axons to the CNS^41^, and our work shows that hemocytes are closely adjacent to and/or form direct contacts with sensory neurons, likely along the neuron cell bodies and dendrites, suggesting the communication involves non-canonical mechanisms. In *Drosophila*, as in vertebrates, signal transfer along all neuronal membrane surfaces, including dendritic synapses and dendrodendritic connections, have been described^42^,^43^, which may also form the interface in neuron-blood cell communication. The transcriptional induction of *Actβ* in response to sensory stimuli recalls previous reports of the transcriptional upregulation of *Actβ* in the formation of long-term memory in both flies and vertebrates^44^,^45^. This suggests parallels between the neuronal regulation within the CNS, and PNS-blood cell circuits, which will be an interesting subject for future study. Based on our findings and another recent report demonstrating that transcriptional regulation of the related *BMP Decapentaplegic (Dpp)* in the *Drosophila* wing epithelium depends on the K^+^ channel *Irk2* (ref. 46), we propose that cellular electrochemical potential may be a more general theme in the expression of TGF-β family ligands.

Our findings in the *Drosophila* model pioneer a new concept that has not been shown in any vertebrate system to date– the neuronal induction of self-renewing, tissue-resident blood cells. These cells correspond to the broadly distributed system of self-renewing myeloid cells that are present in most vertebrate organs, which by lineage are completely independent from blood cell formation fueled by hematopoietic stem cells^3^,^4^,^5^,^6^,^47^,^48^. In vertebrates, TGF-β family ligands such as Activin A and TGF-β regulate the activity and immune functions of macrophages, and cellular and humoral immune responses, in multiple ways through autocrine and paracrine signalling^49^,^50^. While the autonomic neuronal and glial regulation of hematopoietic stem and progenitor cells in the bone marrow has been recognized^51^,^52^,^53^,^54^,^55^, the role of sensory innervation in bone marrow hematopoiesis remains unknown. Even more so, nothing is known about the role of the nervous system in the regulation of the independent, self-renewing myeloid system of tissue macrophages. However, local neurons and sensory innervation of many organs including skin, lung, heart and pancreas^56^,^57^,^58^,^59^ and inducible changes in the self-renewal rates of tissue macrophages^2^, suggest that principles of neuronal regulation are likely also at work in vertebrates, providing a link between neuronal sensing and adaptive responses of local blood cell populations.

## Methods

### Fly strains

*Drosophila* lines used were *HmlΔ-DsRed(2 copies)/CyO* (ref. 4), *Actβ^ED80^/unc13GFP* (ref. 21), *tub-GAL80^ts^* (ref. 60), *Actin5c-GAL4* (Bloomington), *Actβ-GAL4* (ref. 18), *daw-GAL4* (ref. 21), *myo-GAL4* (ref. 13), *Cha-GAL4* (Bloomington), *en-GAL4* (Bloomington), *Sal-GAL4 (GMR85E05-GAL4* insertion in or near *Salm*, Bloomington BN46802); *21-7-GAL4* (refs 4, 41), *repo-GAL4* (ref. 29), *He-GAL4* (ref. 61), *OK6-GAL4* (Bloomington BN64199 (ref. 62)). The line *HmlΔ-Gal4*, *UAS-GFP*; *He-GAL4* was a kind gift of Jesper Kronhamn and Dan Hultmark. UAS lines used were *UAS-CD4-td GFP* (ref. 63), *UAS-mCD8-GFP* (ref. 64), *UAS-Stinger* (Bloomington), *UAS-luciferase* (gift from Katherine Sepp), *UAS-Actβ* (ref. 19), *UAS-babo(CA)* (ref. 11), *UAS-Kir2.1* (ref. 27), *UAS-shi dn^ts^* (ref. 28). UAS-RNAi lines for *dSmad2*, *punt*, *babo*, *Act-β* and *tkv* from TRIP, VDRC and^19^,^65^, in some cases in combination (for example, for Actβ silencing *UAS-Actβ RNAi* 4A2; *UAS-Actβ RNAi* VDRC ID 12174 was used; for *dSmad2* silencing *UAS-dSmad2 RNAi 3A2*; *UAS-Smox RNAi VDRC* was used). Control crosses used yw (Bloomington) or *w^1118^* (Bloomington) as indicated. Other lines used were split GFP *Drosophila* lines from Kristin Scott^9^. *Repo-LexA::GAD* from Tzumin Lee^66^; *Cha3.3kb-LexA::GAD* from Soeren Dieglemann and Matthias Landgraf^67^. Unless noted otherwise, fly crosses were maintained at 25 °C, for *in vivo* RNAi at 27 °C.

### Immunohistochemistry and microscopy

Antibodies used were anti-HRP-FITC (1:500, Jackson) for pan-neuronal staining^68^, mouse or rat anti-elav (1:50, Developmental Studies Hybridoma Bank), rabbit anti-PS4 (1:100, M. Crozatier) for lamellocytes, mouse anti-Lz (1:10, Developmental Studies Hybridoma Bank) for crystal cells, goat anti-GFP (1:2,000, Rockland), rabbit anti-RFP (1:1,000, Rockland), and Alexa fluorophore conjugated secondary antibodies (1:200, Molecular Probes). Hemocytes were released by dissecting larvae in a drop of Schneider’s medium (Gibco, Invitrogen) or PBS. To bleed circulating hemocytes, larvae were opened at the ventral anterior and posterior sides, and hemolymph was allowed to leak out, avoiding to apply pressure. To prevent dislodging of resident hemocytes or contamination with lymph gland hemocytes, larvae were monitored through a fluorescence stereomicroscope. Resident hemocytes were released by opening the remainder of the larva and scraping the body wall with a needle under microscope guidance, avoiding the lymph gland. For the release of total hemocyte numbers, both procedures were combined. Hemocytes were allowed to attach to glass slides for 15–30 min, followed by standard fixation (4% PFA) and immunocytochemistry^4^,^5^. For larval fillet preps, larvae were pinned down on Sylgard plates and ventrally filleted in a drop of PBS. Gut, fat body and trachea were removed, and the fillet was fixed for 20 min in 4% PFA. Fillets were washed in PBS, unpinned, permeabilized, blocked and immunostained according to standard protocols, staining overnight with gentle agitation^4^,^5^. Lymph gland dissections and stainings were performed as described in (ref. 69), using sets of larvae 72–80 h AEL (2.5–2.8 mm). Images were obtained on a Leica DMI4000B, and live larvae and larval fillets were imaged on a Leica M205FA stereomicroscope^4^. Leica SP5 confocal microscopy was used to obtain high-resolution images. In all experiments, identical settings for experiments and controls were maintained, and images were processed in Adobe Photoshop using identical recorded action settings.

### Hemocyte quantification

To quantify resident hemocytes in segmental HPs, blood cells were visualized by fluorescence microscopy and counted by external inspection of intact live larvae, excluding the compact clusters of hemocytes in the terminal segment HP. Total numbers of hemocytes per larva, and percentages of circulating hemocytes were determined by selective release and automated hemocyte quantification using ImageJ^22^. ‘Total Hemocytes’=[number of circulating hemocytes]+[number of resident hemocytes]. ‘% Circulating Hemocytes’=[number of circulating hemocytes] × 100/[total hemocytes]. Unless indicated otherwise, experiments used larvae 72–80 h AEL (2.5–2.8 mm length), that is, late 2nd instar larvae^4^. This was achieved by timed embryo collections and measuring larval size using Leica LAS software. For total hemocyte counts, hemocyte numbers of experiment larvae were normalized relative to background-matched parallel controls. Depending on the individual experiment, baseline counts of matched controls were typically in the range of 2,000 or 3,000 hemocytes; an example of non-normalized counts over a time course can be seen in Supplementary Fig. 2. Hemocyte number averages and circulating hemocyte fraction averages from at least 4–6 larvae per genotype were calculated. The number of larvae n per genotype is listed in the figure legends, with lower numbers typically referring to the control. Phenotypes were confirmed in independent biological replicates. Where applicable, standard deviations and 2-tailed *t*-tests were calculated. *T*-test values correspond to the following: NS (not significant) *P*>0.05; *P≤0.05; **P≤0.01; ***P≤0.001; ****P≤0.0001.

### Hemocyte re-adhesion assay

We used an *in vivo* hemocyte re-adhesion assay^22^. Specifically, hemocytes were dislodged by paint brush manipulation, and the fraction of hemocytes not returning to the resident state was determined after 45 min incubation, using quantitative isolation of circulating and resident hemocyte populations from single larvae^22^.

### Apoptosis and cell proliferation assays

EdU (5-ethynyl-2′-deoxyuridine, Invitrogen) was used at 10 mM for 2 h for *in vivo* feeding experiments, or 1 mM for 2 h in cell culture medium. Hemocytes were released by scraping the larval carcasses, and stained in multi-well cell culture dishes *ex vivo*^4^,^22^. Click-iT EdU proliferation assays (Invitrogen), and TUNEL assays to quantify apoptotic cells (*In Situ* Cell Death Detection Kit, Roche Diagnostics) were performed according to manufacturers’ instructions and used in hemocyte experiments previously^4^. For hemocyte *ex vivo* Actβ stimulation, Actβ was generated by transiently transfecting (Effectene, Qiagen) the plasmid *pAcPA-dActβ*^70^ and a control plasmid (*pAct5C-GAL4* (M.Zeidler)) each into two *Drosophila* cells lines, changing medium after 8 h, and collecting conditioned media 48 h after the previous media change. Hemocytes of *HmlΔ-GAL4, UAS-GFP; He-GAL4* larvae (72–78 h AEL, corresponding to 2.5–2.7 mm) were released in S2 medium (Gibco/Invitrogen). After settling of the hemocytes (∼15 min), medium was replaced by the Actβ conditioned media or control media. Starting 1 h after stimulation, EdU was added for 2 h, and cells were fixed and stained as described above. Fractions of EdU positive cells among GFP positive hemocytes where quantified by Metamorph, maintaining identical settings between stimulated and unstimulated conditions. For *in vivo* EdU experiments, EdU positive hemocytes were quantified by Metamorph and/or manual counting.

### Manipulation of neuronal activity

Genotypes for neuronal silencing experiments were *21-7-GAL4, UAS-mCD8GFP, HmlΔ-DsRed/UAS-Kir2.1; tubGAL80*^*ts*^*/+*, with controls *21-7-GAL4, UAS-mCD8GFP, HmlΔ-DsRed/+*. Endocytosis and synaptic vesicle recycling were blocked by temperature-sensitive dominant-negative shibire in peripheral md neurons (*21-7 GAL4, UAS-mCD8GFP, HmlΔ-DsRed/+; UAS-shi dn*^*ts*^*/+*) with control larvae (*21-7 GAL4, UAS-mCD8GFP, HmlΔ-DsRed/+*). For short term, maximal hyperpolarization, *Kir2.1* or *shi dn ts* expression was reversibly induced at 37 °C (Kir2.1) or 34 °C (shi dn ts) for 30 min and larvae were analysed within an hour. For long term, moderate silencing, larvae were induced at 27 °C for the indicated periods of time from larval hatching onward. For expression of *Kir2.1* and *shi dn ts* in glia as a control tissue, *HmlΔ-DsRed*; *repo-GAL4, UAS-mCD8GFP* line was crossed to *UAS-Kir2.1; tubGAL80*^*ts*^ or *UAS-shi dn*^*ts*^ respectively. Larvae of the genotype *HmlΔ-DsRed/+; repo-GAL4, UAS-mCD8GFP/+*, which did not express the silencing proteins, were included as additional controls. For maximal effects, larvae were induced at 37 °C for 30 min and analysed within an hour after. For all experiments, embryo collections were timed to 12 h or less and embryogenesis was completed at 18 °C before inducing neuronal silencing. Intact larvae were exposed to carbamoylcholine (carbachol), added to water to expose larvae via the cuticle surface in short-term stimulations (at 2.5 or 10 mg ml^−1^), or added to fly food for long-term experiments (at 0.7 mg ml^−1^).

### *Actβ-GAL4* reporter luciferase assay and quantification of GFP expression

Actβ transcription was quantified based on combining *UAS-luciferase* and/or *UAS-CD4-tdGFP* with the *Activin-β* enhancer reporter *Actβ-GAL4*. Larvae were stimulated with carbachol for 2–3 h (see above). Before lysis, larvae were dissected and the anterior portion with the highly *Actβ-GAL4* expressing CNS was carefully removed before lysis. Luciferase levels were quantified using Bright-Glo according to the manufacturer’s instructions (Promega). For quantification of GFP expression, images of controls and carbachol-stimulated fillets were taken at identical settings (Leica M205 fluorescence stereoscope or Leica SP5 confocal). Images were cropped to corresponding regions of identical areas, and image signal intensity was quantified using ImageJ. Signal intensity of carbachol-stimulated samples relative to unstimulated controls was calculated and averaged.

### GFP reconstitution across synaptic partners (GRASP)

The following *Drosophila* genetic combinations were generated and crossed to detect contact between sensory neurons and hemocytes: *lexAop-CD2::GFP11(11-3)/CyO;Cha3-LexA-GAD#4/Tb* and *UAS-CD4::spGFP1-10(4.1), HmlΔ-DsRed/CyO; Pxn-GAL4/Tb*. To observe contacts between glia and hemocytes the following genetic combinations were generated and crossed: *repo-LexA-GAD; lexAop-CD4::spGFP11(11-3)/CyO, P{Wee-P. ph0}2* and *UAS-CD4::spGFP1-10(4.1), HmlΔ-DsRed/CyO; Pxn-GAL4/Tb*. Larvae were filleted and imaged using confocal microscopy.

### Data availability

The authors declare that all data supporting the findings of this study are available within the article and its Supplementary Information files or from the corresponding author on reasonable request.

## Additional information

**How to cite this article:** Makhijani, K. *et al*. Regulation of *Drosophila* hematopoietic sites by Activin-β from active sensory neurons. *Nat. Commun.* **8,** 15990 doi: 10.1038/ncomms15990 (2017).

**Publisher’s note:** Springer Nature remains neutral with regard to jurisdictional claims in published maps and institutional affiliations.

## Supplementary Material

Supplementary Information Supplementary Figures.
