# Supplementary material for: Regulation of Drosophila hematopoietic sites by Activin-β from active sensory neurons
Source: Nat Commun. 2017 Jul 27;8:15990. doi: 10.1038/ncomms15990 (PMC5537569; doi:10.1038/ncomms15990)
Supplement: Supplementary Information — Supplementary Figures. [file ncomms15990-s1.pdf]

Title of file for HTML: Supplementary Information
Description: Supplementary Figures.

Supplementary Figures

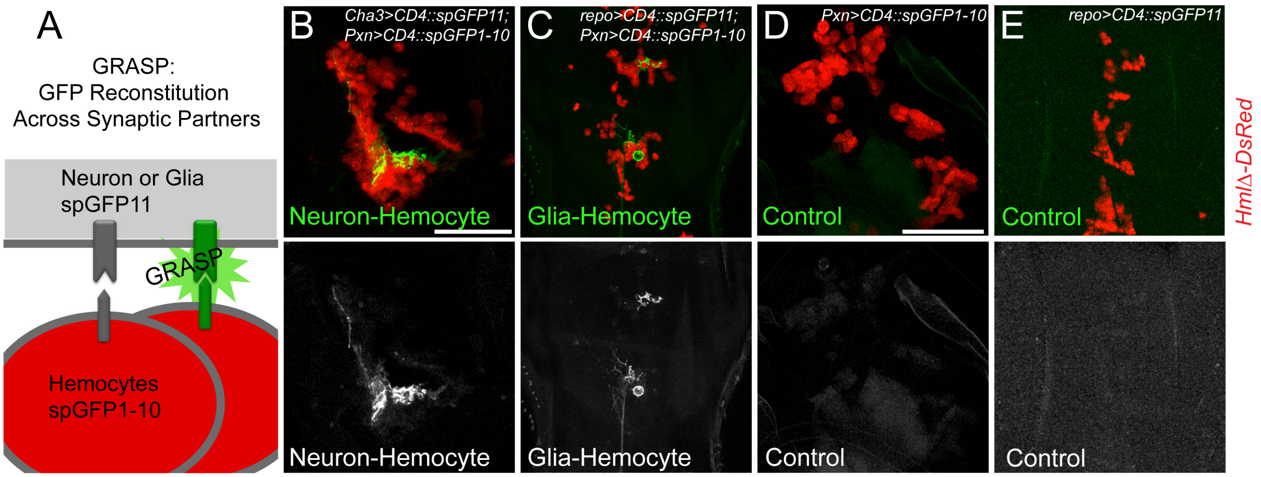

Supplementary Figure 1. GFP Reconstitution Across Synaptic Partners (GRASP). GRASP demonstrates close association between hemocytes and neurons, and hemocytes and glia, in the *Drosophila* larva. (A) Model. (B) Neuron-hemocytes contacts in green, hemocytes in red, lower panel green signal only; genotype *lexAop-CD2::spGFP11/ UAS-CD4:: spGFP1-10, HmlΔ-DsRed; Cha3-LexA-GAD/Pxn-GAL4.* (C) Glia-hemocyte contacts in green, hemocytes in red, lower panel green signal only; genotype *repo-LexA-GAD/+; lexAop-CD4::spGFP11/UAS-CD4 ::spGFP1-10, HmlΔ-DsRed; Pxn-Gal4/+*. (D) control, lower panel green signal only; genotype *UAS-CD4:: spGFP1-10, HmlΔ-DsRed/+; Pxn-GAL4/+*. (E) Control, lower panel green signal only; genotype *repo-LexA-GAD/+; lexAop-CD4::spGFP11/ HmlΔ-DsRed*. Scale bars (B,D) 50 μm.

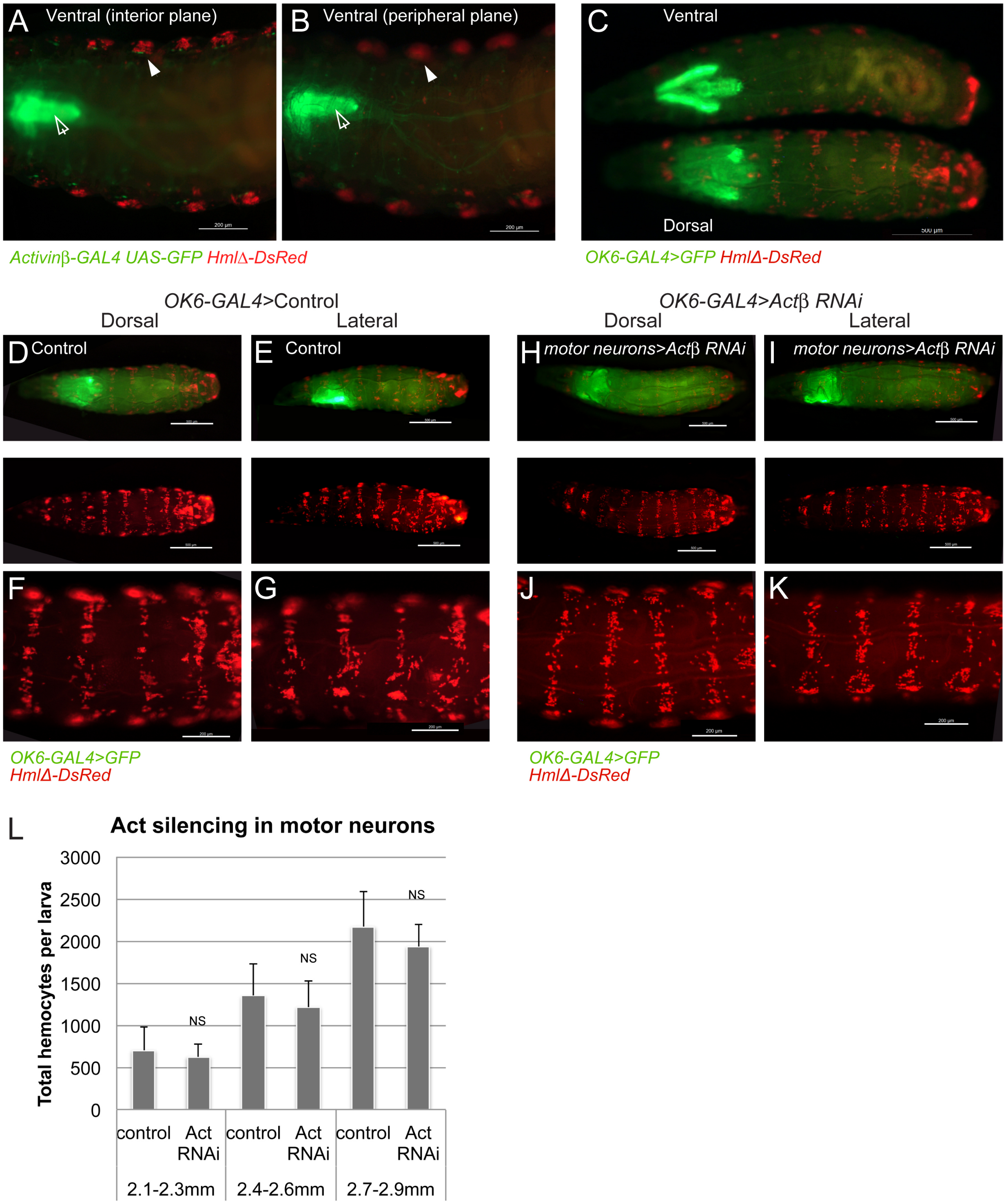

Supplementary Figure 2. Relationship of motor neurons and hemocytes. *Actβ* silencing in motor neurons does not significantly affect hemocytes. (A) *Actβ* expression pattern, *Actβ-GAL4; UAS-mCD8GFP* (green) x *HmlΔ-DsRed/CyO* (red). Ventral view of larva shows *Actβ* driver expression in motor neurons of the ventral nerve cord (open arrowhead), which innervate muscle layers from the interior of the larva; hemocytes clusters (solid arrowhead) are in a distant focal

plane (B) Same larva as in (A), with focus on hemocytes and sensory neurons of the HPs (solid arrowhead). (C) Ventral (top) and dorsal (bottom) view of larvae marked for motor neurons (green) and hemocytes (red), quadrupel recombinant line *OK6-GAL4, UAS-CD8-GFP, HmlΔ-DsRed(2copies)/CyO.* (D-K) Control larva, and *Actβ* RNAi silencing in motor neurons, using the driver *OK6-GAL4*. (D-G) Control, genotype is *OK6-GAL4, UAS-CD8-GFP, HmlΔ-DsRed(2copies) x yw*. (H-K) Motor neuron *Actβ* RNAi, genotype is *OK6-GAL4, UAS-CD8-GFP, HmlΔ-DsRed(2copies)/CyO* x *UAS-Actβ 4A2 RNAi; UAS-Actβ RNAi VDRC12174*. (D, H) Dorsal views, whole larvae. (F, J) same as in (D, H) but single red channel of whole larvae and closeups (lower panels). (E, I) Lateral views, whole larvae. (G, K) same as in (E, I) but single red channel of whole larvae and closeups (lower panels). Scale bars are 500µm, in closeup panels 200µm. (L) Total hemocyte counts per larva, control, and *Actβ* RNAi silencing with motor neuron driver, genotypes as above. Control larvae n=10 for 62-68h AEL (2.1-2.3mm); n=13 for 69-75h AEL (2.4-2.6mm); n=10 for 76-83h AEL (2.7-2.9mm); *Actβ* kd larvae n=14 for 62-68h AEL (2.1-2.3mm); n=13 for 69-75h AEL (2.4-2.6mm); n=7 for 76-83h AEL (2.7-2.9mm). Error bars represent standard deviations, and two-tailed t-test values correspond to NS (not significant) $p>0.05$; * $p \leq 0.05$; ** $p \leq 0.01$; *** $p \leq 0.001$; **** $p \leq 0.0001$.

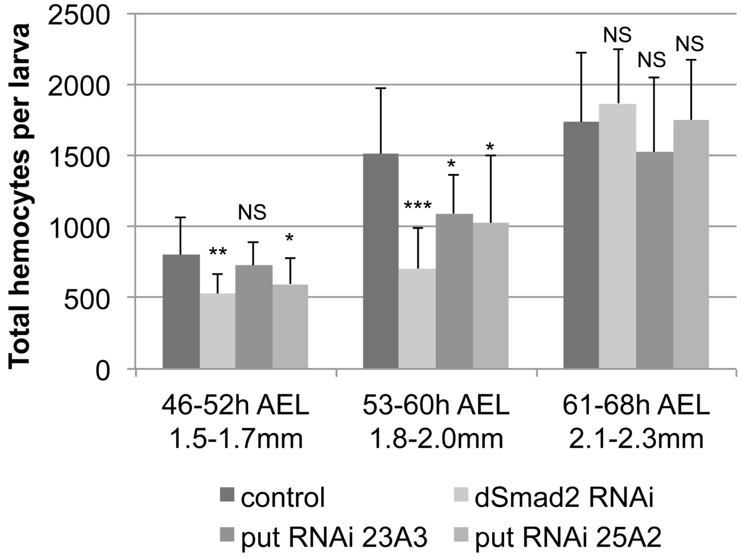

Supplementary Figure 3. Total hemocytes per larva, time course of *dSmad2* or *put* knockdowns in hemocytes. Genotypes are w^{1118}/+; *HmlΔ-GAL4, UAS-GFP/+; He-GAL4/*+ (control); *HmlΔ-GAL4, UAS-GFP/UAS-dSmad2 RNAi 3A2; He-GAL4/UAS-dSmad2 RNAi* VDRC ID14609 or *HmlΔ-GAL4, UAS-GFP/+; He-GAL4/*+ crossed to *UAS-put RNAi (25A2* on 2nd and *23A3 on* 3rd chromosome) (experiments). Note that in young 2nd instar larvae of 46-52h AEL (1.5-1.7mm) and 53-60h AEL (1.8-2.0mm), *dSmad2* or *put* silencing result in reduced hemocyte numbers. However, this effect is compensated in older larvae 61-68h AEL and above (2.1-2.3mm and above) where hemocyte numbers start to overshoot the total numbers of control larvae. Number of larvae as follows: (46-52h AEL corresponding to 1.5 to 1.7 mm): *w1118* (control) n=12; *dSmad2 RNAi* n=13; *put RNAi 23A3* n=14; *put RNAi 25A2* n=13. (53-60h AEL corresponding to 1.8 to 2.0 mm): *w1118* (control) n=11; *dSmad2 RNAi* n=7; *put RNAi 23A3* n=12; *put RNAi 25A2* n=15. (61-68h AEL corresponding to 2.1 to 2.3mm): *w1118* (control) n=15; *dSmad2 RNAi* n=5; *put RNAi 23A3* n=7; *put RNAi 25A2* n=9. Error bars represent standard deviations, and two-tailed t-test values correspond to NS (not significant) p>0.05; * $p \leq 0.05$; ** $p \leq 0.01$; *** $p \leq 0.001$; **** $p \leq 0.0001$.

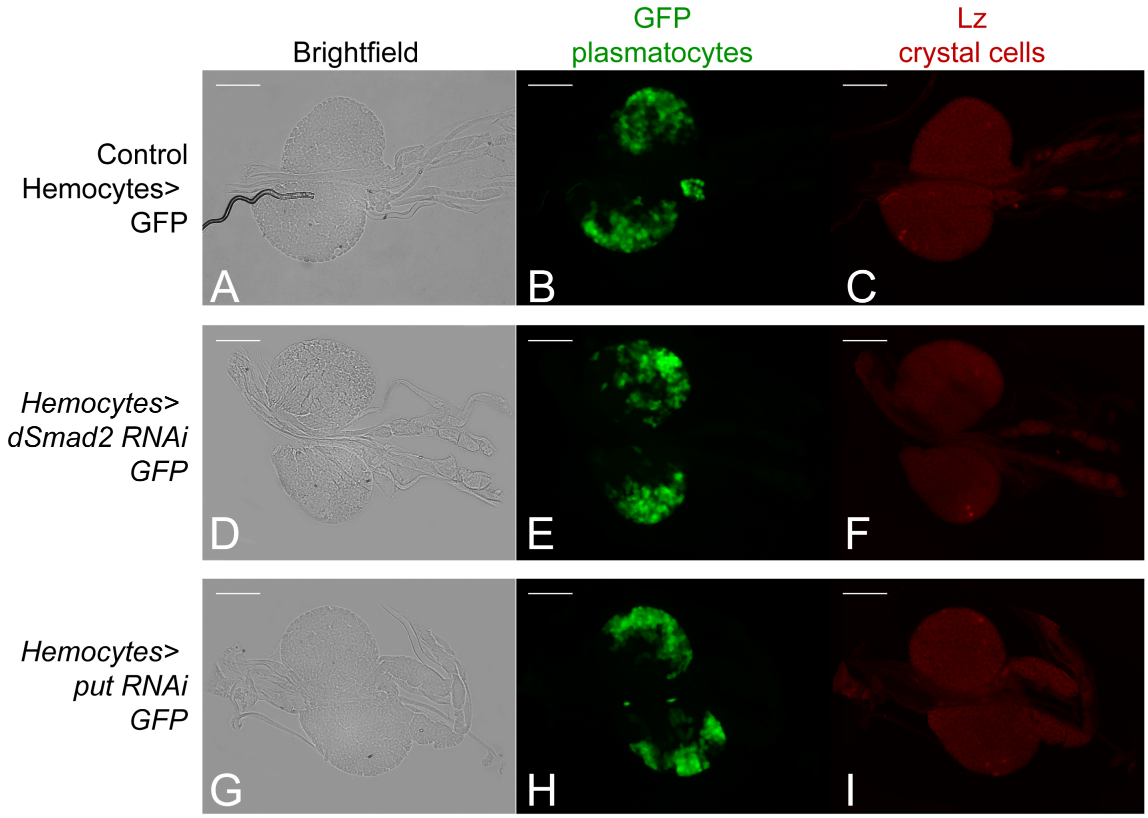

Supplementary Figure 4. Effect of *HmlΔ-GAL4*-driven *dSmad2 RNAi* or *put* RNAi on lymph gland hemocytes. Crosses of driver *HmlΔ-Gal4, UAS-GFP; He-GAL4* with (A-C) Control (*yw*); (D-F) *UAS-dSmad2 RNAi;* (G-I) *UAS-put RNAi 25A2*. Lymph glands of larvae 72-80 h AEL or 2.5-2.8 mm length were dissected, observed under bright field (A, D, G) and stained for GFP (plasmatocytes) (B, E, H) and the crystal cell marker Lz (red) (C, F, I). No significant amounts of lamellocytes were found in lymph glands of the genetic backgrounds examined; antibody (anti-PS4) was verified by overexpression of activated Ras^{V12} to induce lamellocyte differentiation. Scale bars 45μm.

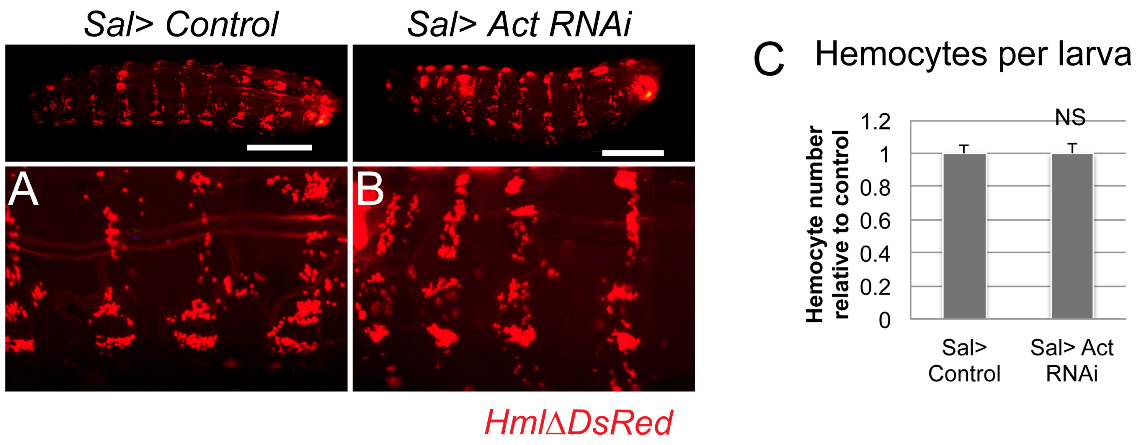

.

Supplementary Figure 5. *Sal-GAL4*-driven in vivo RNAi of *Actβ*. (A-B) resident hemocytes in larvae (2nd instar, 2.5mm), lateral view (upper panel) and close up (lower panel). (A) Control, genotype is *Sal-GAL4, HmlΔ-DsRed/+;, UAS-GFP/+*. (B) Actβ knockdown, genotype is *Sal-GAL4, HmlΔ-DsRed/ UAS-Actβ RNAi* 4A2; *UAS-GFP/UAS-Actβ RNAi* VDRC ID12174 (C) Total hemocytes per larva in Actβ knockdown larvae relative to controls, genotypes as above; n=12 per genotype. Scale bars (A, B) 0.5mm. Error bars represent standard deviations, and two-tailed t-test values correspond to NS (not significant) p>0.05; * p ≤ 0.05; ** p ≤ 0.01; *** p ≤ 0.001; **** p ≤ 0.0001.

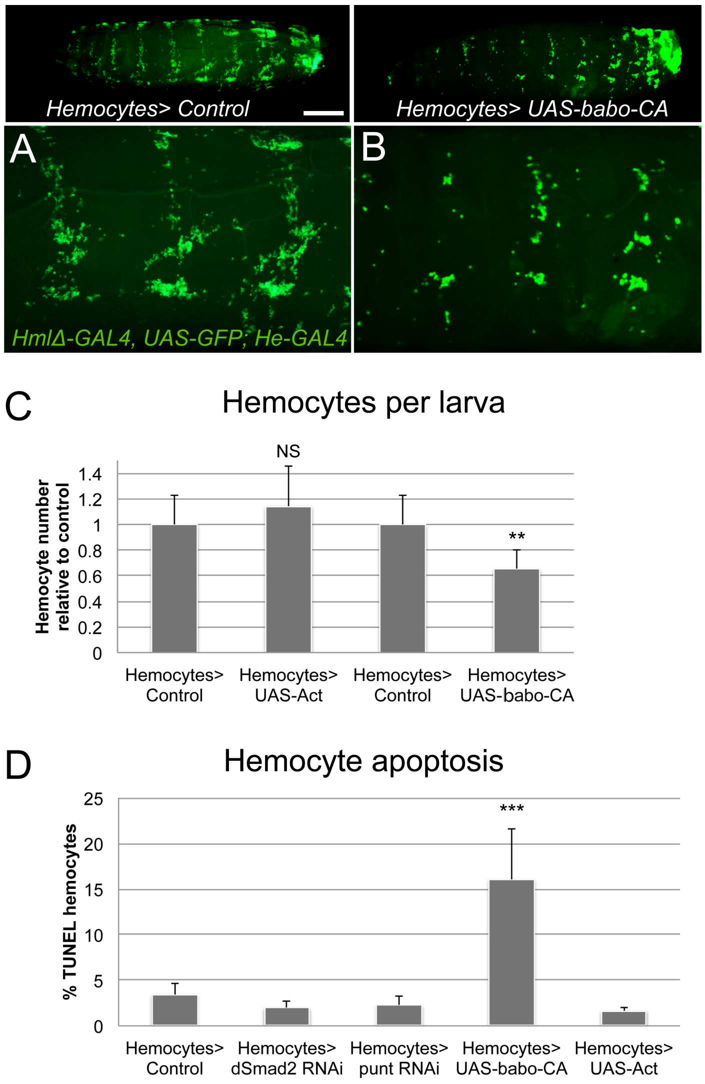

Supplementary Figure 6. Effects of Actβ pathway overactivation in hemocytes. (A-B) Control and overactivation of Actβ/dSmad2 signaling in hemocytes; driver genotype *HmlΔ-GAL4, UAS-GFP; He-GAL4*. Lateral view of whole larvae (upper panels) and close-up (lower panels). (A) Control; (B) *UAS-babo-CA*. (C) Total hemocyte counts per larva, relative to matching control cohorts and genotypes. Genotypes as in (A, B) and cross with *UAS-Actβ* (II); n=6 to 10 per genotype. (D) Apoptosis rates of hemocytes (TUNEL). Genotypes as above and cross with *UAS-Actβ* (III); n=6 to 12 per genotype. Note that high pathway overactivation by *babo-CA* drives hemocytes into apoptosis. Overexpression of Actβ in hemocytes promoted only a mild albeit non-significant increase in total hemocytes, suggesting that certain cell types might be more potent in producing active Actβ ligand than others. No increase in apoptosis was seen upon Actβ overexpression or pathway silencing in hemocytes. Scale bar (A) 0.5mm. Error bars represent standard deviations, and two-tailed t-test values correspond to NS (not significant) $p>0.05$; * $p \leq 0.05$; ** $p \leq 0.01$; *** $p \leq 0.001$; **** $p \leq 0.0001$.

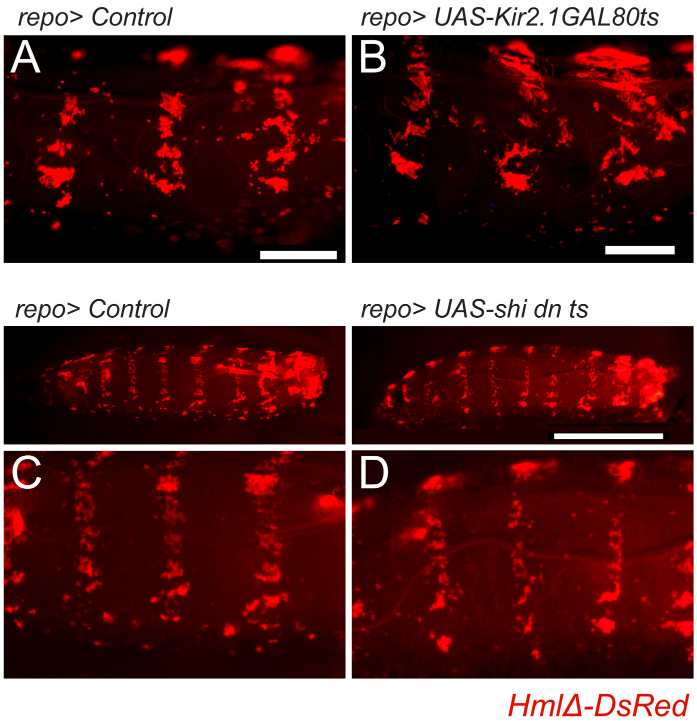

Supplementary Figure 7. 'Silencing' of glia using the driver *repo-GAL4*. As in silencing experiments in PNS neurons, the same transgenes were transiently expressed in glia, and hemocytes were visualized using the reporter *HmlΔ-DsRed* (red). Close-ups of lateral view larvae, in some cases with lateral view whole larvae (upper panels) are shown. (A) Control, genotype is *HmlΔ-DsRed, UAS-mCD8-GFP/+ ; repo-GAL4/ tubGAL80^{ts}*; and (B) Kir2.1 expression, genotype is *HmlΔ-DsRed, UAS-mCD8-GFP/ UAS-Kir2.1; repo-GAL4/ tubGAL80^{ts}.* (C) Control, genotype is *HmlΔ-DsRed, UAS-mCD8-GFP/+ ; repo-GAL4/+*; and (D) shi dn ts expression, genotype is *HmlΔ-DsRed, UAS-mCD8-GFP/+ ; repo-GAL4/ UAS-shi dn ^{ts}*. Scale bars in (A, B) 250µm each, scale bar in (D) 1mm.
